# Predicting Common Audiological Functional Parameters (CAFPAs) as Interpretable Intermediate Representation in a Clinical Decision-Support System for Audiology

**DOI:** 10.3389/fdgth.2020.596433

**Published:** 2020-12-15

**Authors:** Samira K. Saak, Andrea Hildebrandt, Birger Kollmeier, Mareike Buhl

**Affiliations:** ^1^Department of Psychology, Carl von Ossietzky Universität Oldenburg, Oldenburg, Germany; ^2^Cluster of Excellence Hearing4all, Carl von Ossietzky Universität Oldenburg, Oldenburg, Germany; ^3^Medizinische Physik, Medizinische Physik, Carl von Ossietzky Universität Oldenburg, Oldenburg, Germany; ^4^HörTech gGmbH, Oldenburg, Germany; ^5^Hearing, Speech and Audio Technology, Fraunhofer Institute for Digital Media Technology (IDMT), Oldenburg, Germany

**Keywords:** CAFPAs, clinical decision-support systems, machine learning, audiology, interpretable machine learning, precision diagnostics

## Abstract

The application of machine learning for the development of clinical decision-support systems in audiology provides the potential to improve the objectivity and precision of clinical experts' diagnostic decisions. However, for successful clinical application, such a tool needs to be accurate, as well as accepted and trusted by physicians. In the field of audiology, large amounts of patients' data are being measured, but these are distributed over local clinical databases and are heterogeneous with respect to the applied assessment tools. For the purpose of integrating across different databases, the Common Audiological Functional Parameters (CAFPAs) were recently established as abstract representations of the contained audiological information describing relevant functional aspects of the human auditory system. As an intermediate layer in a clinical decision-support system for audiology, the CAFPAs aim at maintaining interpretability to the potential users. Thus far, the CAFPAs were derived by experts from audiological measures. For designing a clinical decision-support system, in a next step the CAFPAs need to be automatically derived from available data of individual patients. Therefore, the present study aims at predicting the expert generated CAFPA labels using three different machine learning models, namely the lasso regression, elastic nets, and random forests. Furthermore, the importance of different audiological measures for the prediction of specific CAFPAs is examined and interpreted. The trained models are then used to predict CAFPAs for unlabeled data not seen by experts. Prediction of unlabeled cases is evaluated by means of model-based clustering methods. Results indicate an adequate prediction of the ten distinct CAFPAs. All models perform comparably and turn out to be suitable choices for the prediction of CAFPAs. They also generalize well to unlabeled data. Additionally, the extracted relevant features are plausible for the respective CAFPAs, facilitating interpretability of the predictions. Based on the trained models, a prototype of a clinical decision-support system in audiology can be implemented and extended towards clinical databases in the future.

## Introduction

Clinical decision-making is a complex and multi-dimensional process which comprises gathering, interpreting, and evaluating data in the context of a clinical case, in order to derive an evidence-based action (1). Due to the complexity of the process, clinical decision-making is obviously prone to errors. Their rates in general practice have been estimated as high as 15% (2). Arguably, wrong clinical decisions can have considerable negative impact on the quality of life of the affected individuals (3). This is also true for decision-making in audiology. Considering that [1] about 5% of the world population and one third of individuals aged above 65 years suffer from disabling hearing loss (4), [2] that the age group above 65 years is the fastest growing population (5), and [3] that decisions are prone to error also in audiology, it is important to continuously improve the precision of clinical decision-making in this domain.

Flaws in clinical decision-making are partly caused by individual differences between physicians with respect to their level of expertise, the subjective nature of the decision-making process, as well as environmental factors. For instance, highly experienced physicians tend to be more accurate in their choice of treatment as compared to novices (6). Furthermore, also experts, similarly to novice physicians, like humans in general, are susceptible to cognitive processing biases. Most often occurring distortions were described as the availability bias, confirmation bias, and premature closure, amongst others (7). Lastly, different physicians may have access to different measurements (data) because different clinics may use different test batteries in their assessment kits which can vary with respect to their measurement precision and validity (8). Additionally, it is possible that in the longitudinal evaluation of a patient, required data from previous potential examinations is missing, or inconsistencies in the administered tests entail difficulties for a physician newly involved in the case (8). In summary, the aforementioned factors arguably lead to variability in the clinical decision-making process across physicians and clinics, and facilitate distortions in diagnostic outcomes. To improve the objectivity, precision and reproducibility of physicians' decision-making, clinical decision-support systems (CDSS) have received an increased attention in many health care domains.

CDSS are information systems that aim to improve clinical decision-making by providing relevant information on relationships between measurements and diagnosis to physicians, patients, or other individuals involved in the clinical context (9). They aim to reduce the information load of physicians by summarizing it through the extraction of patterns and predictions from large amounts of data (10). For instance, physicians can be informed with probabilities of certain medical findings and treatment recommendations, based on imputed case-relevant data which can help to achieve well-informed judgements (9). In addition, CDSS can rule out subjectivity in clinical judgements. Not only can they reduce the impact of processing biases on diagnostic outcomes, but also support novice physicians in their decision-making process to eliminate inter-physician variability in diagnostic outcomes.

The advantage of CDSS has been demonstrated in many previous studies. Just to exemplify with a few, Paul et al. (11) introduced a computerized CDSS for antibiotic treatment. Based on a sample of 2,326 patients in three different countries, the study demonstrated that TREAT improved the hits for an appropriate antibiotic treatment to 70% as compared with physicians who only achieved 57% hits. Another example for a successful CDSS was provided by Dong et al. (12). The authors developed a rule-based CDSS for the classification of headache disorders which correctly identified several types of conditions with an accuracy above 87.2%.

Despite the demonstrated potential of using CDSS, in practice a widespread usage is oftentimes lacking. Developed CDSS may not go beyond the trial stage and physicians may choose not to adopt them (13). Consequently, research has tried to identify potential reasons that lead physicians to refrain from using a CDSS. The Technology Acceptance Model developed by Davis (14) aims to explain this problem of users acceptance with respect to Information Technology in general. It concludes that user's acceptance is influenced by design features, perceived usefulness, and perceived ease of use. The perceived ease of use represents how effortless a system can be adopted and it will causally affect the perceived usefulness. This, in turn, entails how such a system would benefit the user and enhance his or her performance. However, it is believed that physicians may be more prone to assess a system based on trust, rather than its usefulness or ease of use (15). Wendt et al. (16) state that the extent to which users are convinced of the validity of the information provided by the CDSS is crucial for acceptance. On the one hand, this can be achieved by including physicians in the development of such CDSS, by means of interviewing physicians along with extensive piloting. This could lead towards a CDSS that addresses the physicians needs and, additionally, incorporate it in such a way that it fits into the physician's workflow. On the other hand, enabling physicians to understand how the CDSS works may further increase their trust towards them. As a result, physicians evaluate and interpret the system's output and determine its validity, enhancing the level of comfort in utilizing the CDSS (17). Consequently, black box CDSS are rarely accepted, so that understandable algorithms need to be established for achieving physician's trust.

In the medical discipline of audiology, in addition to the aforementioned issues, the heterogeneity of the applied assessment tools among different clinics leads to further challenges in clinical decision-making (8). As a result, comparability in audiological diagnostics and treatment recommendations across clinics is compromised. This in turn may lead to some of the errors that occur in provided diagnostic decisions. Moreover, the differences in applied audiological measures may turn out to pose challenges for the development of a CDSS, aiming to enhance diagnostic precision. This is because data from different measurement sources need to be accounted for and integrated in a CDSS. Thus far, the use of machine learning and CDSSs in the field of audiology is restricted to automatizing audiological measures (18, 19), predicting specific diseases, e.g. vertiginous disorders (20), or for a broad classification of individuals into auditory profiles (21). For instance, Song et al. (18) proposed an automated audiometry based on machine learning that resulted in similar estimates at audiogram frequencies, while requiring fewer samples than the traditional manual procedure. Further, Sanchez Lopez et al. (21) identified four different auditory profiles using unsupervised learning, which differ on the dimension of audibility and non-audibility related distortions and may be used for the development of audiological test batteries. However, to the best of our knowledge, no CDSS was yet proposed aiming to support physicians in their general diagnostic endeavor for a variety of audiological findings.

To address this issue and to work out the relevant constituents of a more generally applicable CDSS in the field of audiology that are transparent to the physicians with respect to their underlying properties, Buhl et al. (8) developed the Common Audiological Functional Parameters (CAFPAs). The CAFPAs aim to represent the functional aspects of the human auditory system in an abstract and measurement-independent way. They can act as an interpretable intermediate representation in a CDSS, i.e. CAFPAs are estimated from audiological measures, and the CAFPAs can be used to infer probabilities of audiological findings or treatment recommendations. In other words, the CAFPAs aim to integrate audiological data from a variety of sources, next to allowing physicians to interpret and validate them. This is achieved through ten different parameters, describing relevant conditions which help to determine hearing disorders (8).

Due to their characteristic of being an abstract representation that does not depend on specific audiological measures, the CAFPAs provide a common framework for physicians, regardless of environmental factors, i.e. differences in audiological measures and clinical expertise. In addition, the CAFPAs were defined in an expert-driven way, through discussions among experts (8) and by considering the statistical analysis performed by Gieseler et al. (22). By including audiological experts into the development process of the CAFPAs, the crucial aspect of users involvement, here physicians, has been addressed. In summary, the need for a CDSS with decision-making steps that become transparent to physicians is addressed by the CAFPA framework aiming to act as interpretable intermediate layer in a CDSS. This property ensures that a future CDSS based upon the CAFPAs will not be a black box.

Buhl et al. (8) already demonstrated the general feasibility of the CAFPAs to be used as abstract representation of audiological knowledge. By an expert survey conducted in the opposite direction as compared with the typical diagnostic process, audiological experts rated outcomes of audiological measures and CAFPAs for given diagnostic cases (i.e., audiological findings as well as treatment recommendations). This resulted in audiologically plausible distributions. As a next step towards a CDSS for audiology, Buhl et al. (23) built a labeled data set in the typical direction of audiological diagnostics, i.e. experts rated audiological findings, treatment recommendations, and CAFPAs based on individual patients' data from audiological measures. The suitability of the given data set as a training distribution for future algorithmic audiological classification tasks was assessed and confirmed. Hence, Buhl et al. (23) provided a data set with expert-derived CAFPAs for given audiological measure data in a sample of individual patients. Based on this data set, machine learning models for the automatic estimation of CAFPAs from audiological measures can now be built and evaluated as a next step towards a CDSS in audiology.

The current study therefore aims at:

1. Predicting expert determined CAFPAs for given audiological measures using machine learning models;
2. Identifying the most relevant features for the prediction of ten different CAFPAs from the audiological measures, in order to ensure the interpretability of the models and increase physicians' future acceptance of automatically derived CAFPAs;
3. Evaluating the potential of the trained models in predicting CAFPAs for unlabeled data i.e., unlabeled patient cases from available databases.

## Method

### Data Set

As outlined above, Common Audiological Functional Parameters (CAFPAs) are intended as intermediate representations between audiological measures and diagnostic decisions in a CDSS. To empirically instantiate CAFPAs, Buhl et al. (23) conducted an expert survey on a data set containing audiological measures (*N*_total_ = 595) provided by the Hörzentrum Oldenburg GmbH (Germany). Thus, given the audiological data, experts were asked to assess CAFPAs, as well as to provide diagnostic decisions for *N*_labeled_ = 240 patients. The remaining data of *N*_unlabeled_ = 355 patients will be used as unlabeled cases for further evaluations of the trained algorithms. With the labeled data set we intend to quantify the link from audiological measures to CAFPAs.

#### Common Audiological Functional Parameters

**Figure 1:**
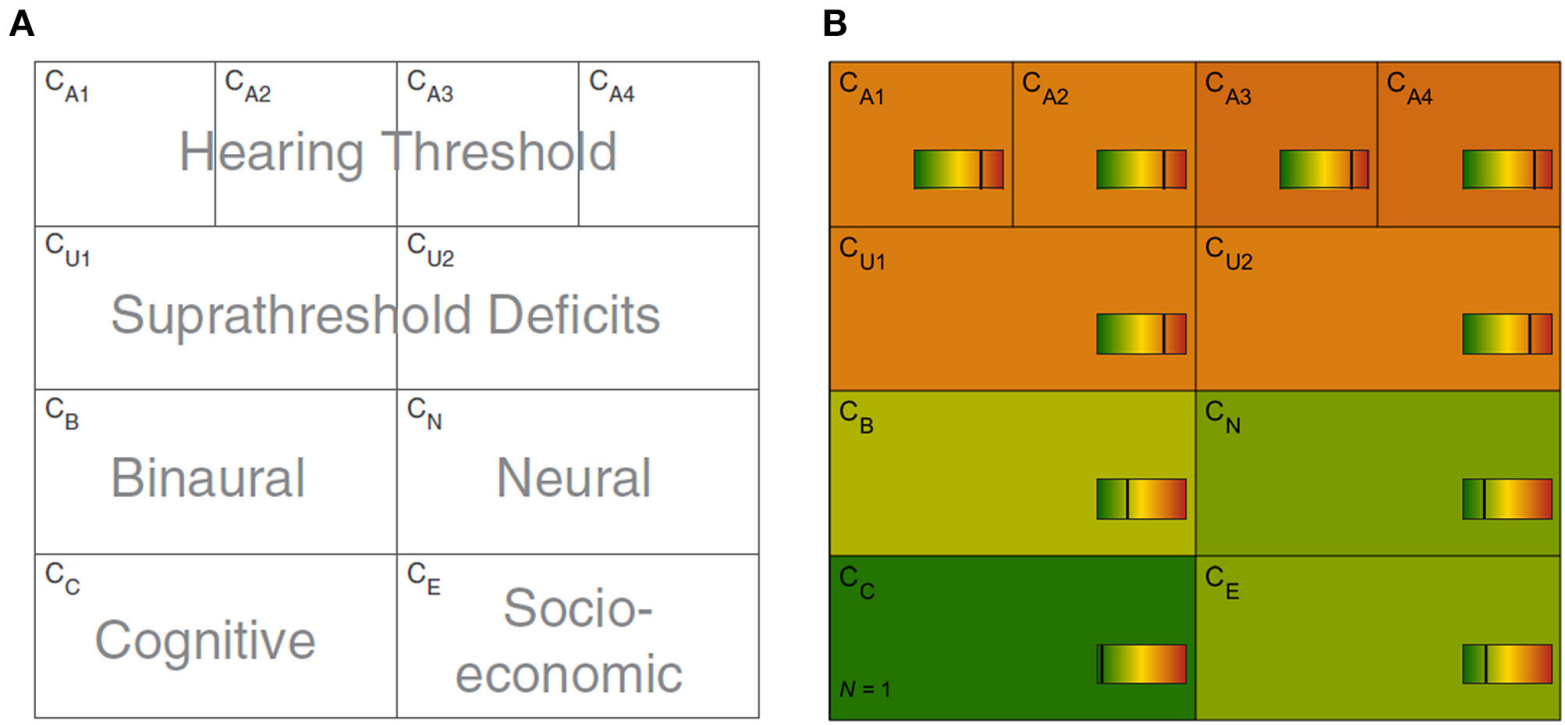
Common Audiological Functional Parameters (CAFPAs). **(A)** Functional aspects of the human auditory system represented by the CAFPAs. **(B)** Exemplary CAFPA representation. The color bar corresponds to the interval [0 1]. The respective value of each CAFPA is indicated by the color of the area, as well as by the vertical line within the color bar.

The CAFPAs describe functional aspects of the human auditory system and are thereby independent of the choice of audiological measures. The covered functional aspects are summarized in Table 1 and Figure 1A.

**Table 1:** Overview and description of CAFPAs.

**Functional aspects**	**CAFPA**	**Description**
Hearing Threshold	C_A1_ C_A2_ C_A3_ C_A4_	The CAFPAs CA1-CA4 refer to the hearing threshold at increasing frequencies. Hearing threshold refers to the minimum sound level that is required to hear a sound. It is indicated as the threshold at which a sound is detected at least 50% of the time. The hearing thresholds are given in decibels of the hearing level (dB HL) for given frequencies in comparison to the normal population. Values between 0 and 20 dB HL are considered to be within the normal range, whereas increasing dB HL values correspond to increasing hearing loss for the given frequencies (24).
Suprathreshold deficits	C_U1_ C_U2_	These components refer to deficits at levels above the threshold (24) for lower (C_U1_) and higher frequencies (C_U2_). Even if hearing threshold levels are within the normal range, deficits may still be present in the suprathreshold range, e.g. with deficits in speech recognition (25).
Binaural hearing	C_B_	Binaural hearing reflects processes taking place in the central nervous system, which enables hearing with two ears simultaneously (24, 26). On the one hand, this entails the ability to perceive different signals that reach the two ears as one, termed binaural fusion (24). On the other hand, binaural hearing allows spatial hearing and sound localization (26, 27).
Neural processing	C_N_	This CAFPA broadly defines the involvement of neural components in the hearing process, such as the cochlear and auditory neurons (24).
Cognitive components	C_C_	Cognitive components play a role in hearing deficits. Studies have widely indicated a correlation between age-related hearing loss and cognitive decline, even though the causal mechanisms remain unclear (28). Cognitive decline may reduce available cognitive resources for auditory processing. Conversely, reduced auditory input caused by hearing loss may lead to a degradation of inputs to the brain, causing cognitive decline. In any case, a strong association between cognitive measures and hearing loss has been found (29).
Socio-economic status	C_E_	This CAFPA contains information regarding the socio-economic status of an individual, which is a combined measure of economic and social status, found to be positively associated with better health (30).

In a CDSS for audiology, the CAFPAs are planned to act as an interpretable intermediate layer. They should be determined from audiological measures. Subsequently, a classification of audiological findings, diagnoses, or treatment recommendations for the provision with hearing devices could be performed based on their basis. The CAFPAs are defined on a continuous scale in the interval [0 1], indicating the degree of impairment. Their scale can be graphically displayed in a traffic-light-like color scheme (cf. Figure 1B), where for the respective functional aspect green [0] represents “normal” and red [1] represents “maximally pathological” status.

#### Expert Survey

The database of the Hörzentrum Oldenburg GmbH (Germany) contains audiological measures, cognitive tests, and self-reports on multiple questionnaires from more than 2,400 patients. Complete data on main variables relevant for the expert survey was available for 595 patients. A detailed description of this database was published by Gieseler et al. (22). In the expert survey by Buhl et al. (23), a part of this database was labeled for the purpose of linking CAFPAs to audiological diagnostics. Thereby, audiological experts were asked to label individual cases from the database. They were asked to indicate expected CAFPA values as well as audiological findings and treatment recommendations on a one-page survey sheet on which the patients' data were displayed in a graphical manner.

The following audiological measures and subjective patients' reports were displayed to the experts. The audiogram (for air and bone conduction), which characterizes the hearing threshold of a patient, i.e. which minimum sound pressure level can be perceived at different frequencies. The adaptive categorical loudness scaling [ACALOS; (31)] which aims to assess the loudness perception of the patient. Furthermore, speech intelligibility was captured with the Goettingen sentence test [GOESA; (32)]. The Vocabulary test [German: Wortschatztest (WST); (33)] was used as a measure of verbal intelligence. Information regarding the socio-economic status was assessed with the Scheuch-Winkler index [SWI; (34)]. The DemTect (35) was selected as a measure of cognitive performance which also serves as a screening measure for dementia. Finally, self-reports on age, gender, first language, the presence of tinnitus in the left/right ear, and hearing problems in quiet and in noise were additionally displayed to the experts.

Experts were asked to indicate expected CAFPA values on a continuous color bar based on their clinical experience in audiology. Furthermore, they had to tick diagnostic cases from a provided list of options. Audiogram and loudness scaling results were available for both ears. If there was an asymmetry between the ears in a given case, experts were instructed to consider only the worse ear for estimating respective CAFPAs and diagnostic classes. According to the above procedure, expert labels were obtained for 240 different patient cases. Out of these, for consistency check, a subset was given to multiple experts. Thus, in total 287 labeled expert survey sheets were available. The mean age of the sample including labeled cases was 67.5 (*SD* = 11.3). For the present analyses, the expert labels provided for the CAFPAs are assumed to reflect the ground clinical truth. They will be denoted as ‘labeled’ CAFPAs in the following.

### Model-Building

CAFPAs, which serve as labels, are defined on a continuous scale, leading to a regression problem to be solved for automatic generation of CAFPA values given the above mentioned audiological data (features) for the patients (data points). The model space of the given regression problem contains the lasso regression, elastic nets, and random forests approaches. These predictors will be applied and evaluated in comparison with regard to the loss function. The model space covers the range between higher interpretability and lower flexibility (lasso regression, elastic net) and lower interpretability and higher flexibility [random forests; see (36)]. The comparative evaluation aims at capturing the well-known trade-off between interpretability and potentially higher predictive performance accuracy, whereby the first is a similarly crucial feature for a CDSS in order to be accepted in applied context.

We use a 10-fold Cross-Validation (CV) in the model-building process. The data set for the prediction of each CAFPA was randomly split into training (80% of the sample, containing the validation set) and test sets (20%). The validation set is used for hyperparameter tuning. In contrast, the test set is not being used in the model-building process, but for evaluating the model with respect to prediction accuracy for future cases.

#### Features and Labels

**Figure 2:**
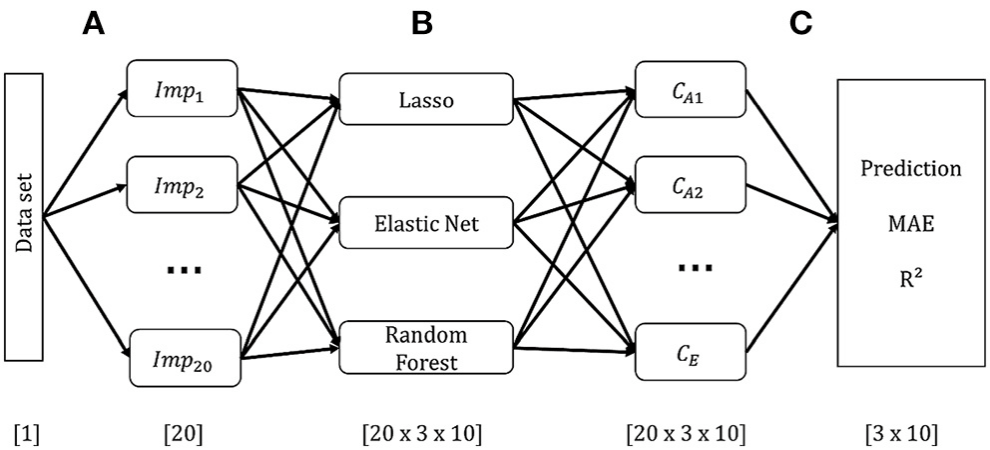
Schematic overview of the model-building pipeline. Numbers in brackets indicate dimensions. **(A)** Pre-processing and generation of 20 data sets based on multiple imputation of missing values. **(B)** Building models on each imputed data set for each CAFPA. **(C)** Prediction of CAFPAs with the three selected models on each imputed data set. Evaluation of prediction accuracy using the Mean Absolute Error (MAE), and *R*^2^ averaged across multiple data sets.

Each of the ten CAFPAs was treated as individual label. Features are the audiological measures as used in the expert survey (Table 2). If an audiological measure includes several measurement variables (e.g., the audiogram is measured for different frequencies), each of these variables is used as feature. In total, 44 features were used for modeling. Corresponding to the instruction in the expert survey to rate CAFPAs for the worse ear in case of an asymmetric hearing loss, only audiogram and adaptive categorical loudness scaling data for the respective worse ear of each patient are included as features. To retain information regarding the asymmetry between ears, an asymmetry score serves as an additional feature. This score reflects the absolute difference in dB between the pure-tone average hearing loss (PTA; audiogram (air conduction) averaged over the frequencies 0.5, 1, 2, and 4 kHz) of the left and right ear [e.g., (38)]. Figure 2 depicts the general analysis pipeline for predicting the CAFPAs.

**Table 2:** Overview of audiological measures and features.

**Measure**	**Number of Features**	**Features**
Audiogram (air conduction)	11	Frequencies: {0.125, 0.25, 0.5, 0.75, 1.0, 1.5, 2.0, 3.0, 4.0, 6.0, 8.0} kHz; worse ear (according to PTA) selected
Audiogram (bone conduction)	7	Frequencies: {0.5, 0.75, 1.0, 1.5, 2.0, 3.0, 4.0} kHz worse ear (according to PTA) selected
Asymmetry score	1	Difference of pure-tone average (PTA) hearing loss for left and right ear in dB
Adaptive categorical loudness scaling (ACALOS)	12	With 1.5 & 4 kHz narrowband noise; worse ear selected – Lcut (juncture point between linear parts of the loudness function) – Mlow (slope of first linear part) – Mhigh (slope of second linear part) – L2.5 (hearing threshold level) – L25 (medium-loudness level) – L50 (uncomfortable level) (37)
Goettingen sentence test (GOESA)	3	SRT (speech reception threshold) Slope SI (speech intelligibility) (32)
Vocabulary test (WST)	1	Sum of correct answers (33)
DemTect	1	Sum score of five tests (08: suspect of dementia; 912: slight cognitive impairment; 1318: normal cognitive behavior) (35)
Hearing problems (HP)	2	quiet; noise 0 (no hearing loss) to 5 (very severe)
Scheuch-Winkler Index (SWI)	1	Sum score for categories profession, education, and income (34)
Age	1	Age in years
Language	1	Native speaker (German); non-native speaker
Gender	1	Male; female
Tinnitus	2	Presence; right and left ear

#### Pre-processing

To avoid statistical dependency due to multiple evaluations of certain patients by multiple experts, for all analyses we randomly selected the CAFPA results of one experts' response only. For all features, but for hearing problems in quiet and noise (74.3%), at least 94.2% of the data were available. Where necessary, we imputed missing data on features by using Multivariate Imputation with Chained Equations [MICE; (39)]. MICE is an approach in which missing values on one feature are estimated based on the remaining features included into the imputation model. Missing values are replaced by predicted values with an added random error term. To minimize potential bias due to one single addition of the random error, the imputation process is repeated multiple times. Imputed values are updated in each iteration, resulting in a given imputed data set. By generating multiple such imputed data sets, MICE accounts for the uncertainty that stems from predicting missing values (39). It is a superior missing data technique as compared with single imputation methods, such as mean or predicted values imputation (40). We used 20 iterations for each imputed data set and generated a total amount of 20 imputed data sets. This amount was shown to be sufficient for successful estimation of the missing data (39, 41). The plausibility of the imputed values was visually inspected across iterations and imputed data sets, as well as through a density plot of the imputed values for each feature. Modeling was carried out on each of the 20 imputed data sets, instead of averaging the data prior to the model-building process (41). Thus, we averaged the predicted CAFPAs after being estimated over multiple data sets.

Missing labels were not imputed. For the prediction of each CAFPA label only those cases were included for which the corresponding CAFPA label was available. In total, 97.5% of the labeled CAFPAs were available. Thus, for each predicted CAFPA, only minor sample size differences occurred.

#### Lasso Regression and Elastic Net

Lasso regression and elastic net are both linear regression models that are closely related to each other. As with linear regression, coefficients are estimated, such that the Residual Sum of Squares (RSS) is minimized. Both lasso regression and elastic net perform feature selection by introducing a penalty for the size of the coefficients (36). By feature selection, a more parsimonious model is being achieved, so that model flexibility and interpretability is optimized. Lasso regression and elastic nets use different penalties. Whereaslasso regression introduces the *l*_1_ penalty (Equation 1), elastic nets combine the *l*_1_ with the *l*_2_ penalty (Equation 2).

(1) $$\begin{array}{rcl} RSS & + & \lambda \overset{p}{\underset{j=1}{\sum }}|\beta_{j}| \end{array}$$

(2) $$\begin{array}{rcl} RSS & + & \lambda \overset{p}{\underset{j=1}{\sum }}\beta_{j}^{2} \end{array}$$

With *l*_1_, the model will penalize the sum of the absolute values of the regression coefficients depending on the tuning parameter λ and thus, sparse models result because coefficients can be shrunken exactly to zero. The size of the selected λ determines the strength of the penalty, with larger values of λ corresponding to a stronger regularization (36). The tuning parameter is being selected by cross-validation in the model-building process (see below).

In contrast, the *l*_2_ penalty does not eliminate coefficients, but shrinks irrelevant features towards zero, next to grouping correlated features together by assigning them similar coefficient sizes (36). Combining both penalties, as in elastic nets, will have three consequences: Irrelevant features will be eliminated, less important features will be shrunken towards zero and correlated features will be grouped together. The relative contribution of each penalty can be fine-tuned with α, a tuning parameter ranging on a scale from [0 1]. As part of the model building process features were standardized for both lasso regression and elastic net, to ensure an equal impact on all coefficients.

For lasso regression, we evaluated λ values that cover the range between the least squares estimate (simple linear regression including all features, λ = 0) to the null model (including no feature and using the mean of the labels as predicted value, λ → inf). The λ value minimizing the loss function of the validation set was selected by means of 10-fold CV separately for each imputed data set.

For elastic net, we performed a grid search of the length 10 for α and λ, using the caret train() function in R. That is, we considered a combination of ten potential values for both α and λ in the grid. Values for α and λ minimizing the loss function on the validation set were selected with 10-fold CV for each imputed data set (cf. Figure 4).

#### Random Forests

Random forests combine multiple decision trees for improving the accuracy and robustness of predictions as compared to those achieved by a single decision tree. Decision trees perform recursive binary splitting of the feature space, that is, a feature that leads to the largest reduction of the RSS is being selected for a split, such that two distinct regions are obtained at every step of the tree building process. In every step, the splitting procedure is repeated based on other features, such that multiple regions in the multivariate space of the observed data are obtained. The prediction is different for each determined region and it corresponds to the mean of the observed response variable in the respective regions. For random forests, multiple trees are built. To avoid building the same decision tree multiple times, only a specified number of features was considered at each split. This enforces different structures of the achieved decision trees and it has the effect of de-correlating the trees before being averaged for the final prediction. As such, the variance of the prediction for future cases (test data) is being minimized (36).

For the current analyses, we tuned the number of features considered at each split (*mtry*) using the tuneRF() function from the randomForest package in R (42). TuneRF() searches for optimal values for *mtry* given the data. The final number of features selected at each split was then determined using the proposed *mtry* values for the 500 trees built for each fold of the 10-fold CV.

### Model Evaluation Based on Labeled Cases

#### Prediction of the CAFPAs

We evaluated the models' performance using the Mean Absolute Error (MAE) as loss function and the coefficient of determination (*R*^2^) between labeled and predicted CAFPA values. As mentioned above, for each of the ten CAFPAs, 20 imputed data sets exist. Accordingly, we built all models (lasso, elastic net and random forest) multiple times on each imputed data set. This resulted in 20 × 3 models for each CAFPA [20 × 3 × 10]. For final model evaluation, we then averaged the MAE and *R*^2^ values across multiple estimations for each CAFPA [3 × 10]. In addition, the correlation between the labeled and predicted values were estimated and plotted. Density plots for labeled and predicted values are provided as well. The null model was chosen as a general baseline to improve upon.

#### Feature Importance

**Figure 3:**
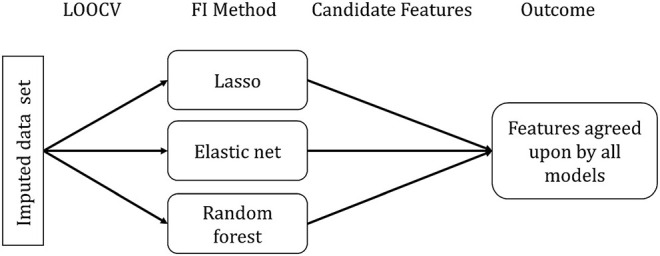
Feature importance (FI) analysis pipeline. FI Method indicates the specific method for each model to extract the relevant features.

For assessing feature importance, we randomly selected one of the 20 imputed data sets, as we did not expect significant differences between the data sets. Furthermore, we did not observe differences when inspecting the standard deviation of the predicted CAFPAs across multiple imputed data set. The selected data set was used to build all three models using Leave-One-Out-Cross-Validation (LOOCV) for each CAFPA [1 × 3 × 10]. LOOCV performs CV by leaving out one observation to be considered as validation set. No additional test set was set aside (differently from the prediction of the CAFPAs), considering that no predictions on future data are made. Figure 3 depicts the feature importance analysis pipeline.

Feature importance assessment is identical for lasso regression and elastic net and it directly follows from the definition of the methods. Due to the different approaches of feature selection that characterize the specific models, selected features differ across models. We used the selection frequency of each feature across all LOOCV models to determine feature importance. Features selected for more than 50% of the LOOCV models are candidate features to be considered relevant.

For each random forest model, we calculated a feature importance measure. For each tree (*n* = 500) in the random forest, 2/3 of the data was used for resampling with replacement. The remaining 1/3 of the data is termed out-of-bag (OOB). Predictions for each data point *i* were made by averaging all trees in which *i* was part of the OOB sample. The loss function can be calculated from the resulting predictions (36). Subsequently, the importance of a given feature *p* was determined by calculating the loss function for each tree in the forest, including all features, next to calculating them with a permuted feature *p*' (36). The average difference between the two loss functions was then normalized and scaled to range from 0 to 100, with 100 being the most important (43). Here, all features with importance values above 50 were considered candidate features. Features selected as candidates by all three models were taken as most relevant features for the prediction of a respective CAFPA.

### Model Application to Unlabeled Cases

Our aim was to obtain a model that allows predicting CAFPAs in the context of a CDSS. Thus, it is crucial that the obtained model(s) are accurate at estimating CAFPAs on unlabeled cases. Therefore, the models were applied to the additional 355 cases (*mean age* = 67.6, *SD* = 12.3) of our data set (*N*_*total*_ = 595) for which no expert labels on CAFPAs are available. To evaluate the predictions on unlabeled cases, we applied model-based clustering (section Prediction of CAFPAs and Clustering for the Unlabeled Data Set). Ideally, we should find the same number of clusters in the CAFPAs predicted by the models from the unlabeled data set, as on the labeled data set.

#### Pre-processing

For the purpose of imputing missings in the unlabeled data set using MICE, we merged this data set with the labeled, previously imputed data set. Because in the future CAFPAs should be predicted for individual cases as part of a CDSS for audiology, potential missing data in single patients will have to be imputed on the basis of larger databases. Thus, merging the unlabeled data set with the labeled one to deal with missingness is in line with procedures suitable for a prospective CDSS. Apart from merging the data sets, the imputation procedure for the features was identical to the one described before. After imputation, we separated the two data sets. In contrast to the model-building analysis, for clustering purposes missing data on CAFPAs were also imputed. However, the imputation was performed exclusively on the basis of the available labeled CAFPAs without considering the features in the imputation model.

To obtain a comparable data set to the labeled one with respect to its size as well as demographic characteristics of the cases (i.e., age, gender, and first language), we applied propensity score matching [PSM; (44)]. The propensity score is defined as the conditional probability that a data point belongs to a treatment group (e.g., in our case to the labeled vs. unlabeled sample) given a set of covariates. It can be estimated by logistic regression (45). Data points with a similar propensity score in the labeled vs. unlabeled data are matched according to the Nearest Neighbor (NN) matching technique (46). NN refers to matching each propensity score from the treatment group (unlabeled data) with the nearest propensity from the control group (labeled data). As a result of the PSM, the unlabeled data set used for unsupervised prediction of the CAFPAs and for subsequent evaluation with model-based clustering consists of 240 cases (*mean age* = 67.4, *SD* = 11.8) that are maximally similar to the labeled cases with respect to demographic features.

#### Prediction of CAFPAs and Clustering for the Unlabeled Data Set

We predicted CAFPAs for the unlabeled cases using the three previously trained models (lasso, elastic net, random forests), each containing 20 models, resulting from the 20 imputed data sets in the model-building part of the present analysis. To evaluate the predictions for unlabeled cases, we applied model-based clustering to [1] the labeled CAFPAs and [2] predicted CAFPAs from the data not containing labels. Model-based clustering assumes the data to stem from a mixture of gaussian distributions, where each cluster *k* is represented by a cluster specific mean vector μ_*k*_ and a covariance matrix Σ_*k*_ (38). The covariance matrix determines the shape, volume, and orientation of the clusters (e.g., varying or equal shape, volume, and orientation). Thus, to determine the most suitable number of clusters for given data, model-based clustering applies different parameterizations of the covariance matrix for different numbers of components [see (47) for the different parameterizations of the covariance matrix]. Accordingly, multiple clustering models can be compared with regard to their properties (i.e., covariance structure and number of components) and the best fitting model selected for the cluster analysis. Model selection can be performed by means of the Bayesian Information Criterion (BIC), which evaluates the likelihood of the model given the data and parameterization, with larger BIC values indicating better fit of a model (48).

To select the optimal model and number of clusters for the data set including labeled CAFPAs, we inspected the BIC to choose the parameterization of the covariance matrix. Thereafter, we determined the optimal number of clusters via visual inspection of the resulting average CAFPA patterns for each cluster. That is, the largest number of clusters differentiating labeled CAFPA patterns was selected (cf. Supplementary Figures 6, 7). As the clusters exist in a multidimensional space, i.e. the ten CAFPA dimensions, we applied principle component analysis (PCA) to visualize the clusters. PCA is a dimensionality reduction method that linearly combines features to result in a new set of orthogonal principle components (PCs). The PCs are ordered with regard to variance, i.e. the first PC explains the largest amount of variance in the data (49). This allows a visualization of clusters in a 2D space (PC1 and PC2), while retaining a large amount of variance existing in the data (50). We then intended to reproduce the same number of clusters of CAFPAs estimated, in the unlabeled data set using the same covariance parameterization, for the purpose of providing comparability between labeled and predicted clusters.

## Results

### Model Evaluation Based on Labeled Cases

#### Model-Building

**Figure 4:**
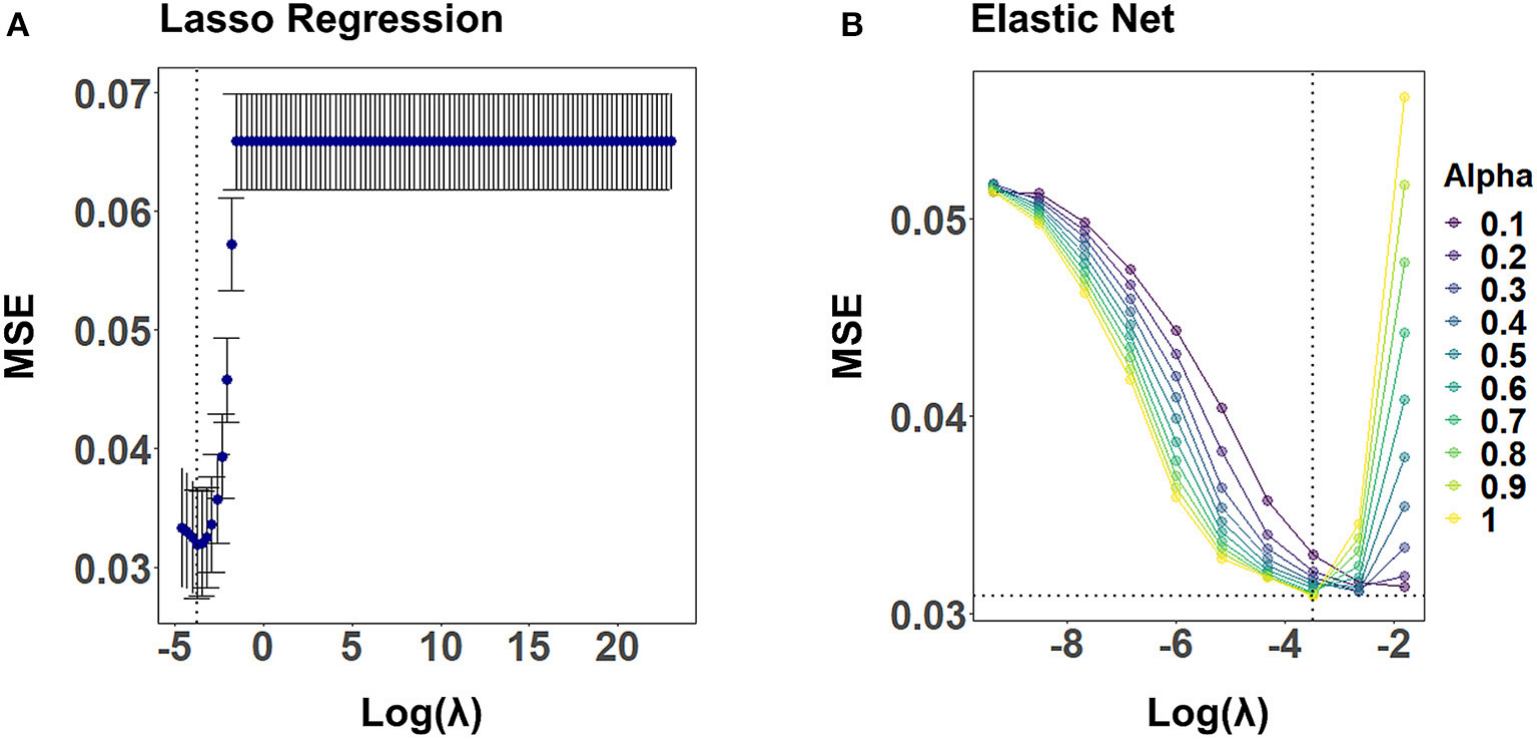
Hyperparameter selection for lasso regression and elastic net using 10-fold CV, exemplarily for C_A2_ and a randomly selected imputed data set. Both plots display the mean-squared error (MSE) as a function of log(λ). The dotted line indicates the values leading to the smallest MSE. **(A)** Tuning of λ for lasso regression. The standard error of λ across CV-folds is shown. **(B)** Tuning of λ for different levels of α with elastic net.

**Figure 5:**
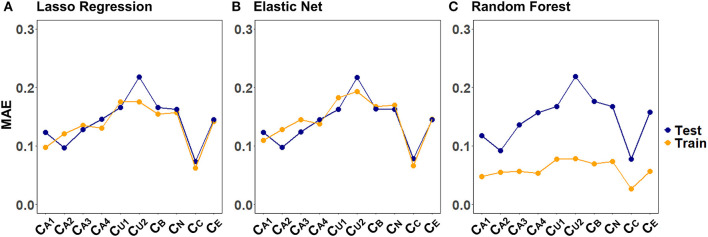
Training and test set loss function (MAE) across CAFPAs for the three models **(A)** lasso regression, **(B)** elastic net, and **(C)** random forest. MAE values correspond to a randomly selected imputed data set.

Figure 4 illustrates the CV results from tuning λ for lasso regression, as well as α and λ for the elastic net, exemplarily, for C_A2_ of a randomly selected imputed data set. Values for α and λ were selected that lead to the largest error reduction in the validation set, as indicated by the dotted line. The results for the remaining CAFPAs for the given imputed data set are provided in the Supplementary Figures 1, 2. Figure 5 depicts the MAE of the trained models for the training and test set across CAFPAs, in comparison to the MAE of the null model. The performance of the lasso regression and the elastic net is comparable. The test error for random forest is slightly higher as compared to the training error but not yet indicative of overfitting.

#### Prediction of CAFPAs

**Figure 6:**
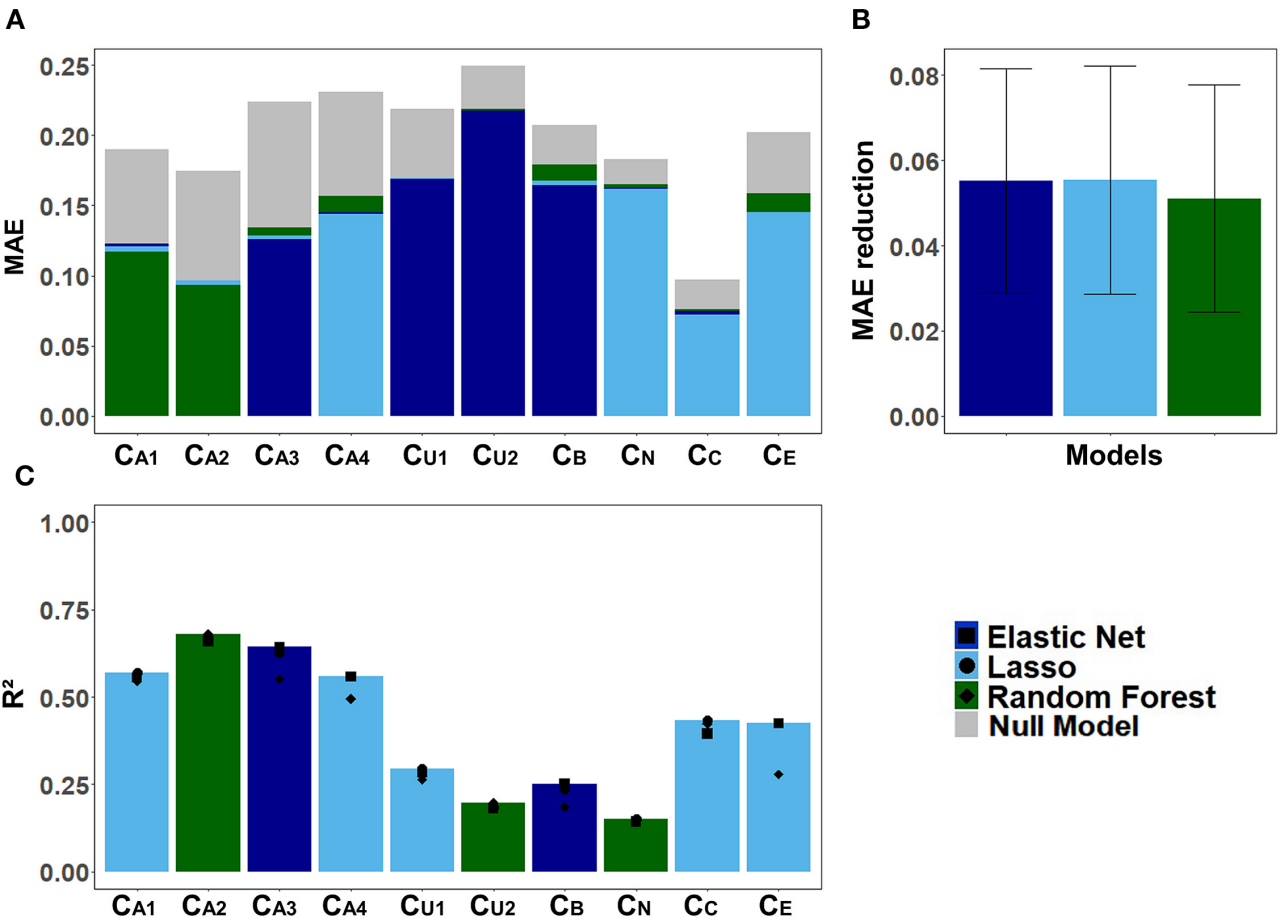
Model-specific predictive performance accuracy for the CAFPAs on the test set, averaged over multiple imputed data sets. Different models are color-coded. **(A)** Mean absolute error (MAE) for each CAFPA. indicates the predictive performance of the null model, and the foremost bar color denotes the model with best predictive performance. **(B)** Mean and standard deviation of the MAE reduction as compared to the null model, averaged over CAFPAs. **(C)** Coefficient of determination (*R*^2^) for each CAFPA. The depicted bar color indicates the model with the best predictive performance. The symbols denote the performance of the respective comparison models.

Figure 6 displays the models' performance at predicting the CAFPAs. In case of all three models, the predicted CAFPAs in the test set were averaged over the imputed data sets. Figure 6A shows the mean absolute error (MAE) between labeled and predicted CAFPAs for the three models as compared with the null model. Although different models perform best for different CAFPAs as indicated by the color bars, the performance across models is comparable, and all models improve upon the null model. The average reduction of MAE over CAFPAs is also similar for the different models (Figure 6B), with the random forests performing slightly worse.

Figure 6C shows the coefficient of determination (*R*^2^) for labeled CAFPAs in the test set. In line with the MAE results, the plot indicates that the performance of lasso regression, elastic net, and random forests was very similar. However, the random forest performed slightly worse for some CAFPAs (C_A3_, C_B_, C_E_). In comparison over CAFPAs, larger differences in predictive performance occurred. The audiogram-related CAFPAs C_A1_-C_A4_ were predicted best, while performance accuracy was lowest for the suprathreshold CAFPA C_U2_ and the neural CAFPA C_N_.

**Figure 7:**
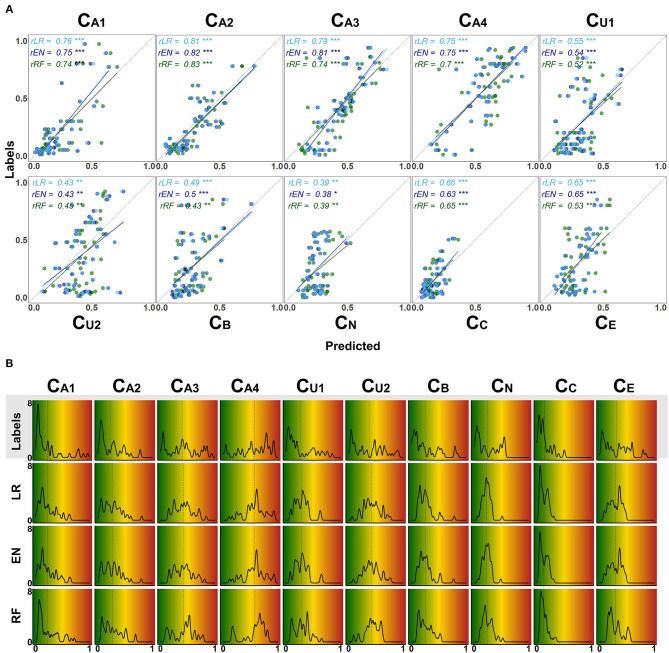
Detailed overview on model performance for different CAFPAs. LR (lasso regression); EN (elastic net); RF (random forests) **(A)** Predicted vs. labeled CAFPA values (for the test set, averaged over imputed data sets) for all CAFPAs. The three models are color-coded. The Pearson correlation *r* and corresponding linear associations are depicted in the corresponding color. The level of significance *p* is indicated by *, **, ***, for *p* < 0.05, *p* < 0.01, *p* < 0.001, respectively. The dotted line indicates perfect prediction. **(B)** Absolute frequency density plots (bandwidth = 0.015) for the, ten CAFPAs. Different rows depict the distributions of the labeled CAFPAs, next to the distributions achieved by the three models. The *x*-axis indicates the CAFPA values; the *y*-axis the absolute frequency of the labeled CAFPAs and those predicted by the respective model. In the background, the color codes corresponding to the CAFPA interval [0 1] are depicted (cf. Figures 1B, 9).

With Figure 7 we provide a more detailed view on the models' predictive performance for different CAFPAs. The scatter plots (Figure 7A) indicate the labeled vs. predicted CAFPAs for individual patients. In addition to the depicted correlations, the range of the labeled and predicted CAFPA values with regard to the interval [0 1] is being visualized in the plot. Except for the neural CAFPA C_N_ and the cognitive CAFPA C_C_, all labeled CAFPAs cover the complete range of potential values. The predicted CAFPAs for all three models generally cover a smaller range of potential CAFPA values, that is, very high values are rarely predicted by the models. Only for the audiogram-related CAFPAs C_A2_-C_A4_ both labeled and predicted values span the complete interval [0 1].

The range of the predicted CAFPAs is further visualized in Figure 5B. Frequency density plots for all CAFPAs are depicted for labeled and predicted values. The labeled CAFPAs are generally distributed over the whole interval [0 1], with a tendency towards lower (green) values especially for the CAFPAs C_A1_, C_A2_, C_U1_, and C_C_ which characterizes the expert ratings, but also the underlying audiological data. To conclude on a sound prediction of CAFPAs, in addition to a high correlation between labeled and predicted CAFPA values and an overlapping value range of the two, the shape of the predicted CAFPA distribution should be similar to the one of the labeled CAFPA scores (see Figure 7B). For most CAFPAs and models, the label distributions are well reproduced by the distributions of the predicted CAFPA scores. Differences between models are smaller than differences between CAFPAs. The strongest similarity between labeled and predicted scores is obtained for the audiogram-related CAFPAs C_A1_-C_A4_. However, the distributions for C_N_ and C_C_ are limited to a restricted CAFPA range as compared with the label distributions. For example, the two maxima of the label C_N_ distribution are not covered by the distributions of the predicted scores.

#### Feature Importance

**Figure 8:**
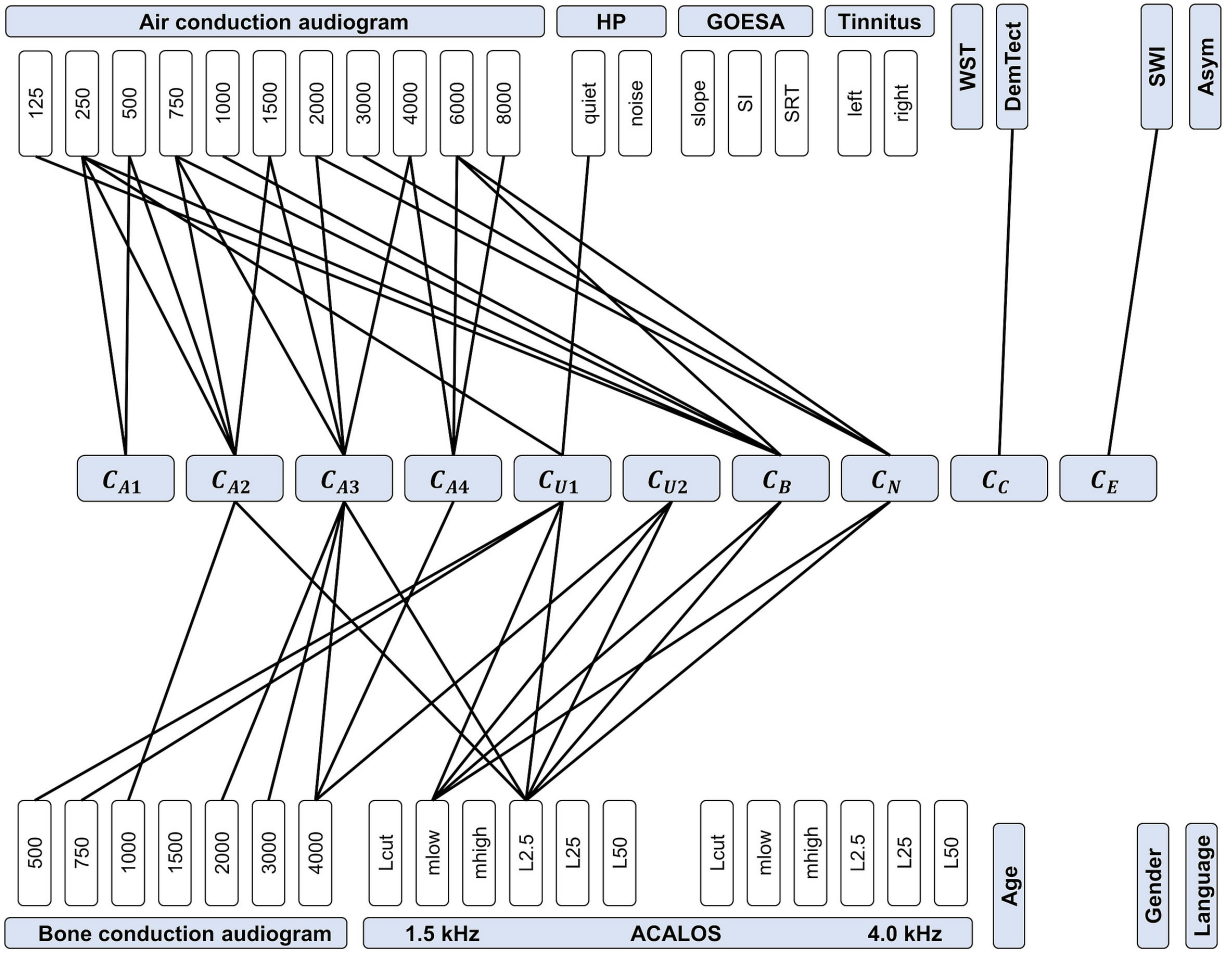
Feature importance for predicting CAFPAs. CAFPAs are displayed in the center of the figure; features in the upper and bottom parts. Measurement parameters are represented by white boxes (cf. Table 1 for abbreviations and units). Black lines displayed as connections between the measures and the CAFPAs indicate features selected by all three models to be relevant for the prediction of the respective CAFPA.

For all models, we assessed feature importance using Leave-One-Out-Cross-Validation (LOOCV). Figure 8 provides a summary of the most relevant features for predicting the different CAFPAs. All features (audiological measures) included in the data set (cf. Table 2) are represented in the plot, and those measures that were selected as relevant features by all three models are connected with the respective CAFPA. The candidate features for each model separately are provided in the Supplementary Figures 3–5.

The most important features for the audiogram-related CAFPAs C_A1_-C_A4_ are air and bone conduction audiogram for plausible frequencies, i.e., frequencies that increase over the four CAFPAs defined for different frequency ranges. For the cognitive CAFPA C_C_ and the socio-economic CAFPA C_E_, the models agreed on only one respective feature, namely DemTect and the Scheuch-Winkler-Index, respectively. In contrast, the selected features for the suprathreshold CAFPAs C_U1_ and C_U2_, as well as the binaural CAFPA C_B_ and neural CAFPA C_N_ are more widely distributed over different audiological measures. Some audiological measures such as ACALOS at 4.0 kHz or tinnitus, and demographic information as well as the asymmetry score were not selected by all of the models for any CAFPA as relevant features, but at least by one model (see Supplementary Figures 3–5).

### Model Evaluation Based on Unlabeled Cases

**Figure 9:**
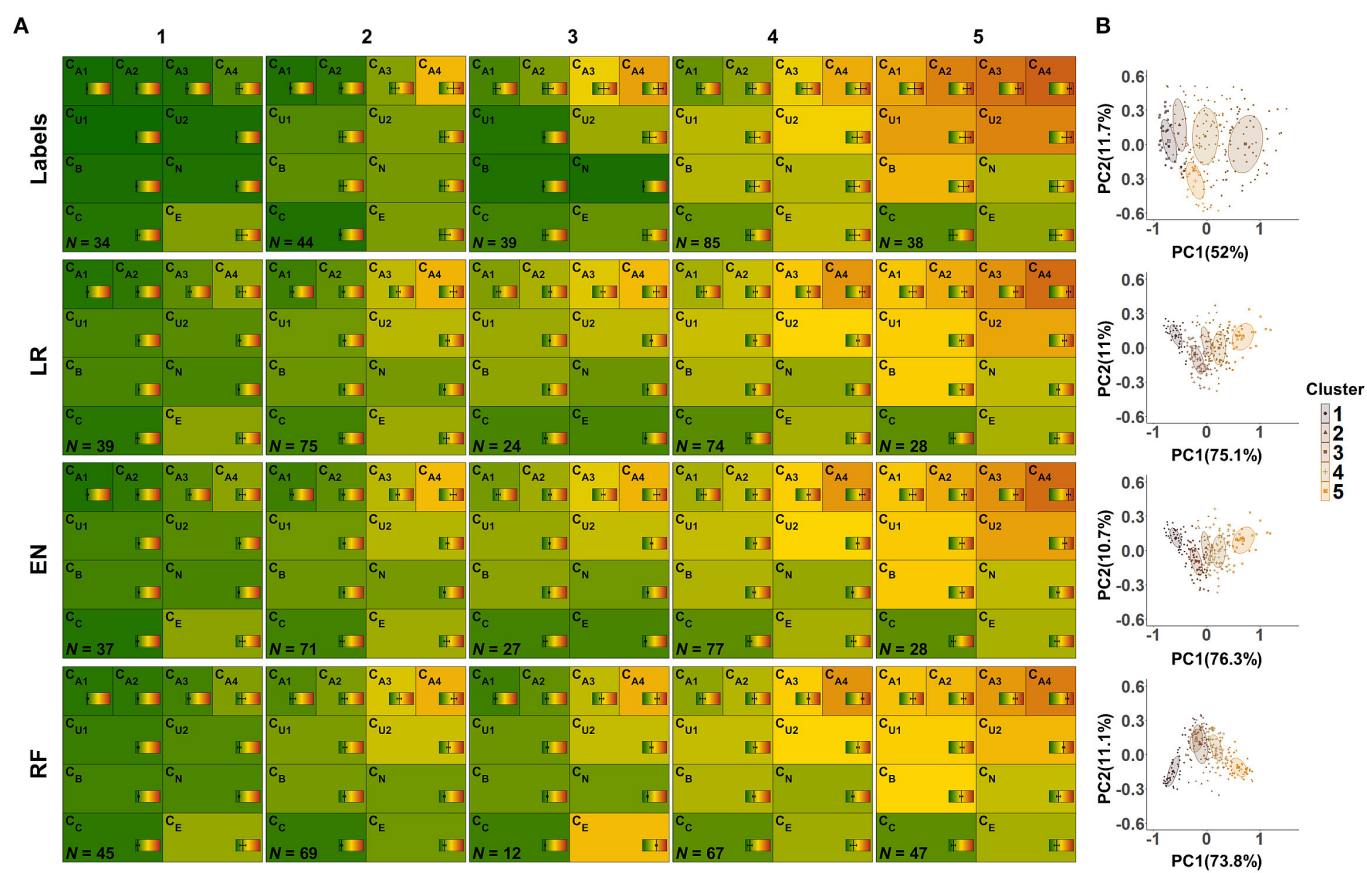
CAFPA clusters estimated using model-based clustering. Rows depict the clusters for labeled CAFPAs (labeled data set), as well as the predicted CAFPAs for matched, unlabeled patients, originating from the models lasso regression (LR), elastic net (EN), and random forest (RF), respectively (unlabeled data set). **(A)** CAFPA patterns for five estimated clusters (columns). The average CAFPAs assigned to each cluster are depicted by the color of the respective area as well as by the vertical line in the color bar. Standard deviations are depicted by horizontal lines in the color bars. *N* indicates the number of patients assigned to each cluster. **(B)** Combined representation of the five estimated clusters. For visualization purposes, the clusters are displayed in the plane given by the first and second principal component (PC) estimated for the respective CAFPA data. The percentage of explained variance by PC1 and PC2 is indicated on the respective axis. Different colors of data points and two-dimensional Gaussian distributions correspond to the different clusters in the columns of **(A)**.

Next, we applied the three models to unlabeled cases for the purpose of investigating the feasibility of predicting plausible CAFPAs also for unlabeled cases. This is an important step toward a CDSS for audiology. Model-based clustering was then used to estimate distinguishable clusters in the ten-dimensional CAFPA data. According to a combination of visual inspection and the BIC, the labeled CAFPAs were best characterized by five clusters using the model λ_k_A_k_ with the identifier VVI. Accordingly, the distribution of the covariance matrix Σ_k_ is diagonal, with varying volume and shape, and an orientation aligned with the coordinate axes (51). Six clusters with the same covariance parameterization reached a marginally higher BIC value (BIC = 1698.8) as compared to five clusters (BIC = 1695.3, Supplementary Figure 6). The additional cluster, however, mainly leads to a separation of the healthy patients into two clusters with higher and lower values for the socio-economic CAFPA C_E_ (Supplementary Figure 7). As separating healthy patients solely on socio-economic status is undesirable, we argue for using five clusters for further analysis. We then applied the same clustering method to the CAFPAs for the 240 matched, unlabeled cases which we predicted using the previously trained lasso regression, elastic net, and random forest. The obtained clusters are depicted in Figure 9A using the typical CAFPA representation that was introduced and used in Buhl et al. (8, 23). Figure 9B additionally displays a combined representation of the five clusters for assessing how well the clusters can be distinguished.

From the left to the right, the labeled CAFPA patterns (labeled data set; first row of Figure 9A) indicate an increasing degree of hearing loss which is expressed by increasing average CAFPA values. The largest differences between the clusters occur for the audiogram-related CAFPAs C_A1_-C_A4_. In comparison to the CAFPA distributions published in Buhl et al. (23), the obtained clusters are in line with normal hearing (cluster 1), different degrees of high-frequency hearing loss (cluster 24), and a more severe, broadband hearing loss (cluster 5). The corresponding plot in Figure 9B shows five distinguishable clusters.

The clusters for predicted CAFPAs on unlabeled cases using the three models (unlabeled data set; second to fourth row in Figure 9A) show CAFPA patterns that are very similar to the labeled CAFPA patterns. However, different numbers of cases were associated to the different clusters, with generally more patients allocated to the clusters with lower CAFPAs. The largest deviation in terms of patients' allocation frequency occurred for random forest, where cluster 5 includes more patients, but on average with less severe hearing loss. This is consistent with the generally lower CAFPA values that the models predicted, in contrast to labeled CAFPAs (cf. Figure 7B). Clusters 2 and 3 for random forest are very similar, with the main difference in the socio-economic CAFPA C_E_. Cluster 3 only contains 12 patients, which is also visible in Figure 9B. In general, similar clusters were obtained for the three models, i.e. the models agreed on the cluster allocation for most of the cases. The agreement between lasso regression and elastic net amounts to 96% and for both lasso regression and random forests and elastic net and random forests to 68%. Further, this similarity becomes evident in Figure 9B), where clusters are displayed on a similar plane in the dimensions of the two first principal components, i.e., PC1 and PC2 are explaining similar amount of variance. In contrast to the clusters for lasso regression and elastic net, the clusters for random forest are depicted with opposite sign with respect to PC2, which is however the same due to symmetry of principal component analysis. Here, the clusters 2 and 3 overlap considerably.

## Discussion

The present study proved the feasibility of automatically predicting Common Audiological Functional Parameters (CAFPAs) from audiological measures. For developing a clinical decision-support system (CDSS) using CAFPAs as interpretable, intermediate representation of audiological knowledge, the automatic prediction of CAFPAs comprises the last step towards a full working first prototype of such a system. We predicted CAFPAs on the expert-determined data from Buhl et al. (23) using lasso regression, elastic net, and random forests. Interpretability of the model predictions was assessed by feature importance measures, and the potential of predicting CAFPAs for unlabeled cases was evaluated using model-based clustering.

### Prediction of CAFPAs

The three models worked reasonably well in predicting the CAFPAs, even though optimal predictive performance cannot yet be achieved. One reason is the limited amount of available data, especially in the range of hearing deficits, and second the choice of the models to some extent. That is, due to the small number of available labeled clinical cases, it was plausible to start with rather simple models to avoid overfitting. As soon as more data becomes available, model flexibility and complexity could be increased, and the here trained methods can be further evaluated to determine which of them turns out to be optimal for CAFPA prediction within a CDSS. Given the available data, the prediction accuracy of the three models was similar, while larger differences occurred between the different CAFPAs, i.e. not all CAFPAs were equally well predicted.

One explanation for performance differences among CAFPAs could be that some CAFPAs are more directly related to the audiological measures than others. This aspect is further discussed in the next section, where we turn to feature importance. A second explanation may be that experts more strongly agree when labeling some of the CAFPAs. Especially given a continuous scale, experts' ratings can be expected to differ from each other to some extent. For example, a meta-analysis of inter-rater reliability on performance status assessment in cancer patients indicated good agreement between raters for about half of the studies; the other half achieved only low to moderate agreement (52). Another study investigated the inter- and intra-rater reliability of audiologists in the estimation of hearing thresholds in newborns, using auditory brainstem response (53). The intra-class correlation of 0.873 was concluded to be satisfactory. However, this value indicates that differences between raters exist. Thus, labels provided by experts, as in the current study, may introduce some bias themselves, although Buhl et al. (23) qualitatively found a good agreement among experts for two reference cases which were given to multiple experts. Such experts' biases, in turn, could lead to less optimal predictions for some of the CAFPAs by using statistical models. To account for these biases and to measure the extent of error introduced by experts, future studies are needed to generate labels by multiple experts for the same cases.

### Model Interpretability via Feature Importance Assessment

By analyzing feature importance, we gained crucial insights into the model-building process as well as into the relationships between audiological measures and CAFPAs. Without exception, all models selected audiologically plausible features for predicting different CAFPAs. This means that the automated generation of CAFPAs could be demonstrated to build upon similar audiological measures like physicians are expected to use in their decision making. Thus, the differences in predictive performance of the models for different CAFPAs (cf. section Prediction of CAFPAs) can be assumed to be due to the measures contributing to the respective CAFPA, as indicated by feature importance. For example, the threshold-related CAFPAs C_A1_ and C_A2_ are among the best-predicted ones. These are closely related to the audiogram (8). For predicting them, the models selected suitable audiogram frequencies, as well as the hearing threshold level L2.5 at 1.5 kHz from the adaptive categorical loudness scaling (ACALOS). In contrast, the CAFPAs that were not as well predicted (e.g., neural CAFPA C_N_; binaural CAFPA C_B_) may be more vaguely related to the measures. That is, impairment in the neural and binaural domain cannot be directly inferred from a single audiological measure, but rather from a combination of audiological measures. Thus, for these CAFPAs, additional measures that better characterize the respective functional aspect need to be included in future test batteries.

In several regards, assessing feature importance contributes to interpretability of the decision-making process. In model-building, it gives access to information with respect to features which were selected by the model. Thereby, it also allows analyzing how experts derived the CAFPAs in the current study, as well as characterizing the data set itself. In addition, being provided with audiological measures (as input of the model) and the derived CAFPAs (output), physicians may be able to understand and trust the automatized generation of the CAFPAs in a CDSS. Therefore, feature importance also helps to achieve physicians trust towards the diagnostic system and could ensure the physician about the validity of decisions provided by the model. Both are crucial for enhancing acceptability and for reinforcing future implementations of an audiological CDSS into the clinical routine (15, 16). The models considered in this study all belong to “intrinsically interpretable” models according to Jung and Nardelli (45), that is, the selected features directly provide interpretability to the experts. However, if in the future more complex models are used, explanations of model predictions that are most informative to specific users could be constructed using the probabilistic model described in Jung and Nardelli (45).

Additionally, by demonstrating that the CAFPAs can be predicted by plausible audiological measures, assessed by commonly used test batteries, here, we provide further empirical support for the concept of the CAFPAs as an abstract representation of the human auditory system. That is, machine learning models were generally capable to learn the underlying relation between audiological measures and the CAFPAs. This is especially relevant for future applications of a CDSS employing the CAFPAs, since predictions in the medical field need to be grounded on available knowledge in the given domain to avoid flawed predictions (54). For instance, in Cooper et al. (55) a neural network predicted low or high risk of in-hospital mortality for pneumonia patients. Subsequent studies analyzing feature importance, however, have revealed that the model assumed asthma to be a protective factor, even though in reality the opposite is true. The prediction error was caused by asthma patients being more carefully treated, due to their higher mortality risk (56). Clearly, this example highlights the importance of the interpretability of predictions within a CDSS in general, and together with the presented results it demonstrates the benefit of the interpretability of the CAFPA predictions that we could achieve in this work. Based on our hitherto available results on CAFPAs, physicians can be provided with the audiological measures that are most influential for the respective CAFPA prediction. As a next step towards a CDSS for audiology, it will be of interest to further enhance interpretability, i.e. by providing physicians with the exact proportions of measurement importance.

### Model Evaluation on the Unlabeled Data Set

A future CDSS would have to be applied to unlabeled cases. Thus, it must be possible to evaluate if plausible CAFPAs can be predicted for unlabeled cases. For this purpose, we applied the trained models on a demographically matched data set of cases for which no labeled CAFPAs were available. Subsequently, we applied model-based clustering on the predicted CAFPAs and obtained five distinguishable clusters that resemble the clusters contained in the labeled CAFPAs.

In clinical practice, different audiological findings occur, such as cochlear hearing loss related to inner ear dysfunction, conductive hearing loss related to middle ear dysfunction, or central hearing loss related to impaired transmission of neuronal signals to the brain. As the data set used in this study consists of a rather small number of clinical cases, it seems plausible that not all audiological findings are well represented in the data set. In particular, the most frequent cases in the current data set are high-frequency hearing loss patients, broadband hearing loss patients, and normal hearing individuals. Thus, the five clusters represent the most frequent audiological findings in the underlying data set well, including different degrees of hearing loss (23). Consequently, it can be assumed that collecting a sufficient amount of more severe clinical cases for additional audiological findings would allow differentiating more clusters.

The performance differences between models for different CAFPAs are reflected in the resulting clusters, as these models were used for the prediction of the CAFPAs for unlabeled cases. If prediction accuracy can be improved in the future for certain CAFPAs, e.g., by including larger data sets and more measures, the separation of audiological findings by the clustered CAFPA patterns will further improve. However, already with the current prediction accuracy, plausible and distinguishable patterns were demonstrated.

Finally, assessing the obtained clusters using the graphical representation of CAFPA patterns, which was introduced by Buhl et al. (8), allows for direct comparability of audiological findings, and it contributes to interpretability of the CDSS by providing a visualization of the functional aspects which describe the group of patients belonging to the respective cluster.

### Clinical Decision-Support System Using CAFPAs

On the way of setting up a CDSS using CAFPAs as interpretable, intermediate layer, the current study closes the gap towards a CDSS working with the input data from a single patient: The prediction models trained here can be used in the future to automatically generate CAFPAs, based on which a classification of audiological findings can be performed. The classification performance could be compared to the classification performance based on the labeled CAFPAs from the expert data set (57).

Most potential for improving toward a testable CDSS lies in applications of the here described models and their extension to larger clinical databases in the model-building process. This is because currently we obtained different performance for different CAFPAs. The analysis of feature importance revealed that the CAFPAs were backed up by different amounts of appropriate audiological measures. Hence, data sets are needed that contain a higher number of patients for all clinically relevant audiological findings, which are characterized by a test battery with information about all functional aspects covered by CAFPAs. In addition, feature importance analysis could also be used in the future to identify redundant audiological measures contained in test batteries used in clinical settings.

For the purpose of integrating data from different clinical test batteries comprising different audiological measures, the CAFPAs act as abstract representation and data standardization format which is independent from the exact choice of measures. Especially data from electronic health records (EHR), i.e. digitally available data from different clinics, could be easily integrated as training data, if CAFPA labels are available for at least some of them. Expert-based estimations of CAFPAs are arguably the most time-consuming. Our future aim is to estimate CAFPAs by a combination of algorithmic generation and expert-coding. For example, experts could confirm and revise automatically estimated CAFPAs instead of labeling each patient case based on audiological data alone.

## Conclusion

In the current study, we applied three modeling approaches, lasso regression, elastic net, and random forests, for the prediction of Common Audiological Functional Parameters (CAFPAs). As all three models provide similar predictive performance, currently all appear suitable choices for an algorithmic prediction of the CAFPAs. We demonstrated that it was possible to estimate CAFPAs as intermediate layer in a clinical decision-support system for audiology, that is, as abstract and interpretable representation for potential users of a CDSS for audiological decision-making.

In line with the aim of setting up an interpretable CDSS for audiology, different aspects provide interpretability to the future users of the tool. First, the CAFPAs themselves act as interpretable representation of audiological knowledge which is independent of the exact choice of measurements, that is, the user can assess the functional aspects that are responsible for the classification of a certain audiological finding. Second, the analysis of feature importance helps the user to reproduce which measures are influential to the estimation of CAFPAs.

Finally, the reported cluster analysis allowed assessing CAFPA prediction performance on unlabeled cases. This is an important property to be covered in a future CDSS. The achieved cluster similarity between labeled and predicted CAFPAs revealed that the trained models generalize well to unlabeled cases, which could also be visually assessed by the CAFPA patterns. Building upon previous work by Buhl et al., the present work is a substantial step towards a CDSS for audiology. However, the models still need to be applied and evaluated on new, larger and more variable clinical data sets in the future. Interpretability needs to be always maintained, even if the models described here might become more flexible when tuned and applied to future data.

## Data Availability Statement

The data analyzed in this study is subject to the following licenses/restrictions: According to the data usage agreement of the authors, the datasets analyzed in this study can only be shared upon motivated request. The analyses scripts can be found here: http://doi.org/10.5281/zenodo.4282723. Requests to access these datasets should be directed to Mareike Buhl, mareike.buhl@uni-oldenburg.de, Samira K. Saak, samira.kristina.saak@uni-oldenburg.de.

## Author Contributions

MB provided the data. SS conducted the data analysis which was continuously discussed with all authors. SS and MB drafted the manuscript and all authors contributed to editing the manuscript. All authors conceptualized and designed the study.

## Conflict of Interest

The authors declare that the research was conducted in the absence of any commercial or financial relationships that could be construed as a potential conflict of interest.
