# Supplementary material for: Predicting Common Audiological Functional Parameters (CAFPAs) as Interpretable Intermediate Representation in a Clinical Decision-Support System for Audiology
Source: Front Digit Health. 2020 Dec 15;2:596433. doi: 10.3389/fdgth.2020.596433 (PMC8521966; doi:10.3389/fdgth.2020.596433)
Supplement: Supplementary file 1 [file Table_1.DOCX]

Supplementary Material

##
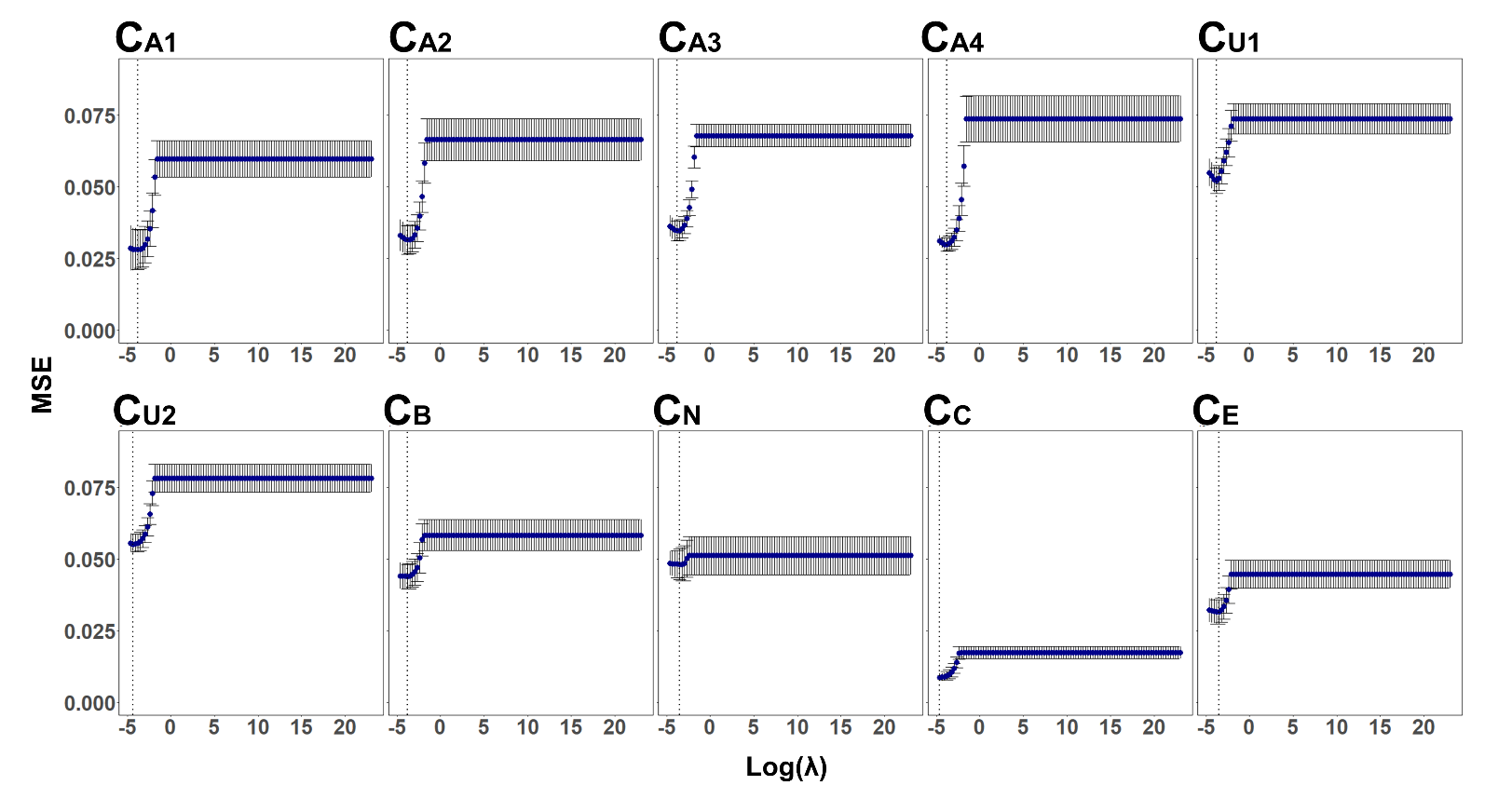
Supplementary Figures

**Figure S1**. Hyperparameter tuning for the ten CAFPAs with lasso regression, exemplarily, for a randomly selected imputed data set using 10-fold CV. The dotted line indicates the λ value leading to the smallest mean-squared error (MSE), which was used for further model training. The standard error of λ across CV-folds is shown. The plot for C_U2_ is the same as shown in Figure 4A.


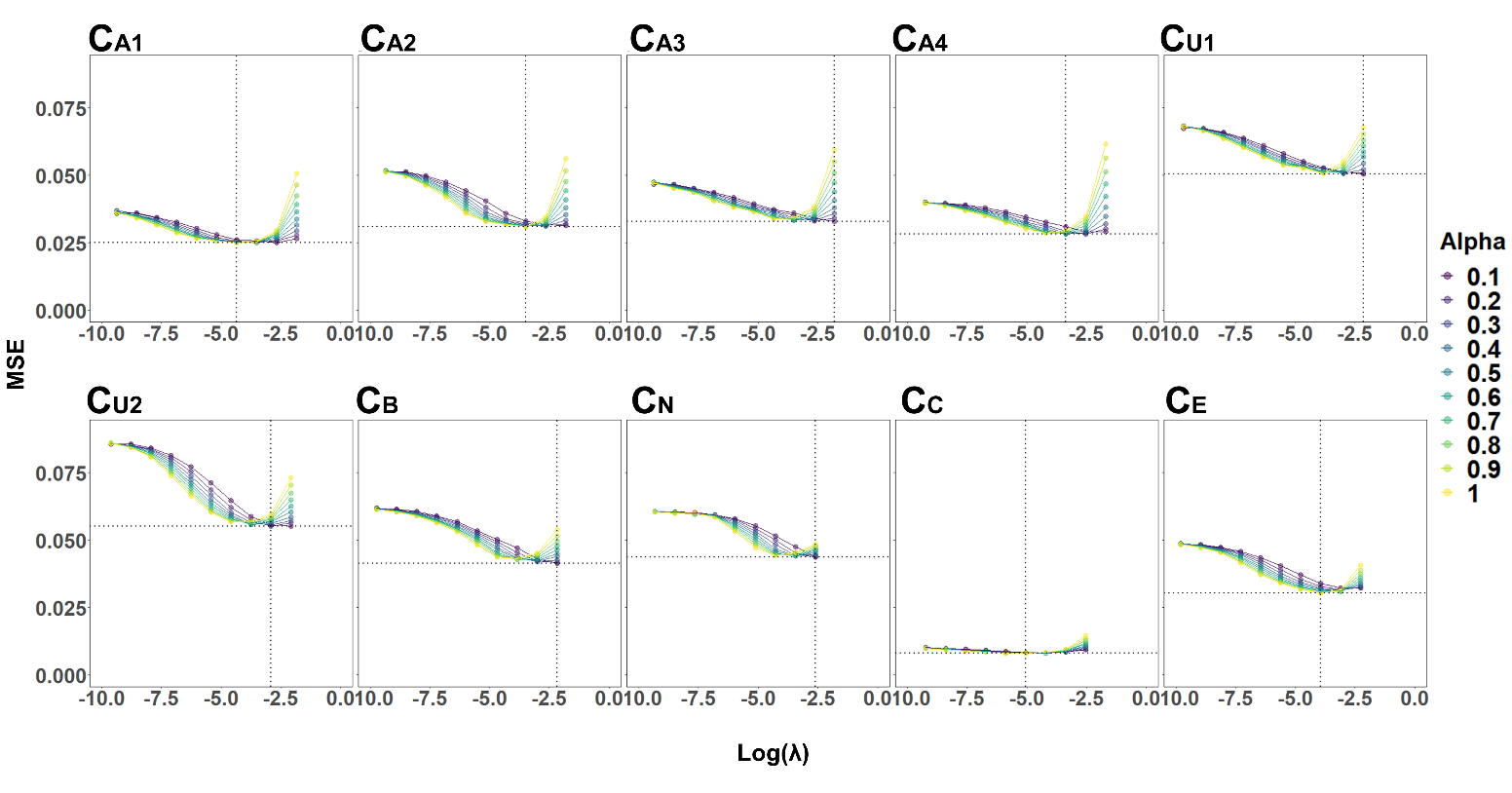
**Figure S2.** Hyperparameter tuning for the ten CAFPAs with elastic net, exemplarily, for a randomly selected imputed data set using 10-fold CV. The dotted lines indicate the λ value of the corresponding α-level leading to the smallest mean-squared error (MSE), which was used for further model training. The plot for C_U2_ is the same as shown in Figure 4B.


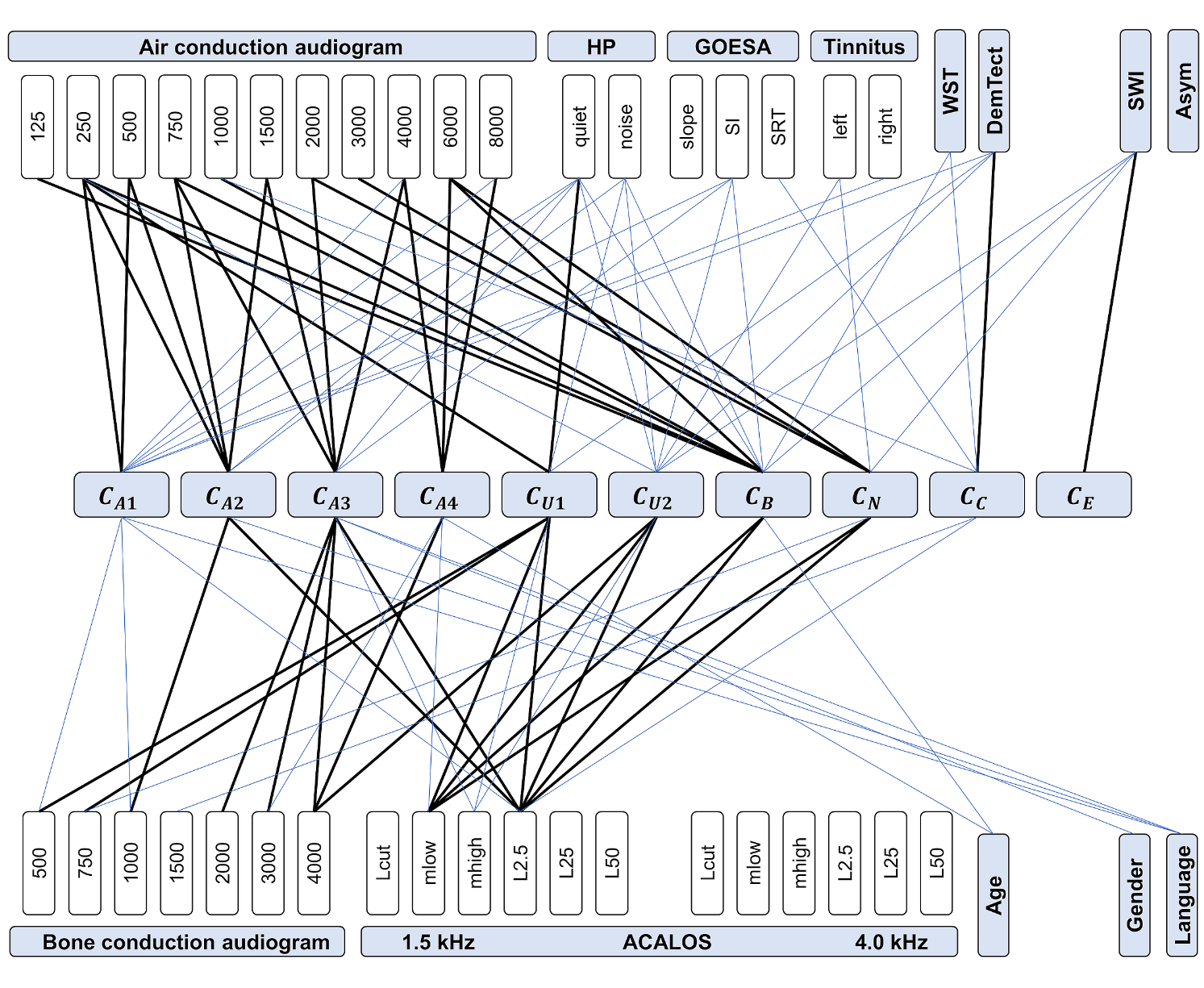
**Figure S3.** Feature importance for predicting the CAFPAs with lasso regression. The CAFPAs are displayed in the center of the figure; Features in the upper and bottom parts. Feature parameters are represented by white boxes. Lines displayed as connections between the measures and the CAFPAs indicate features selected to be relevant for the prediction of the respective CAFPA. Black lines indicate features selected to be relevant by all three models; blue lines indicate the additional features that were selected by lasso regression to be relevant.


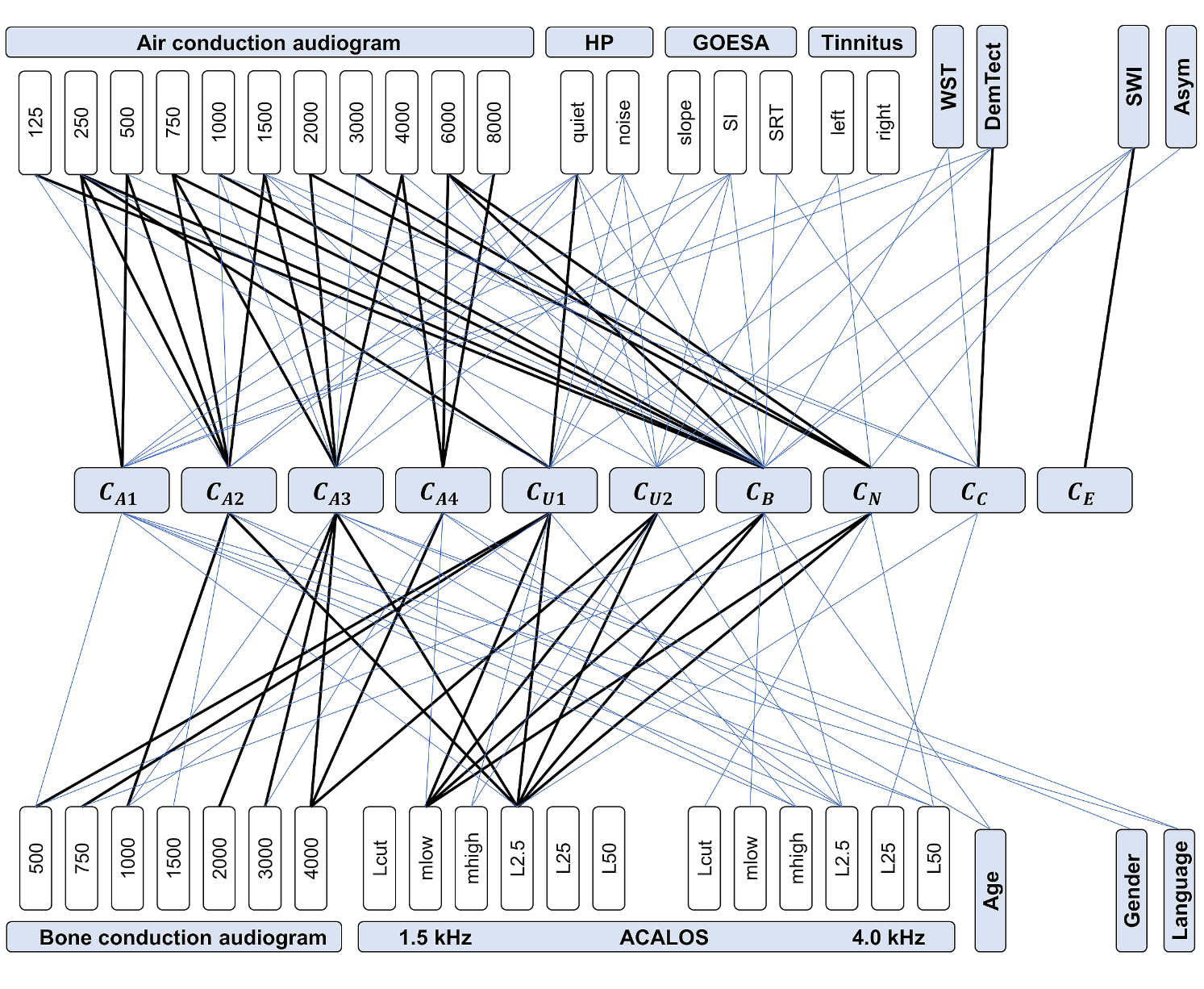
**Figure S4.** Feature importance for predicting the CAFPAs with elastic net. The CAFPAs are displayed in the center of the figure; features in the upper and bottom parts. Feature parameters are represented by white boxes. Lines displayed as connections between the measures and the CAFPAs indicate features selected to be relevant for the prediction of the respective CAFPA. Black lines indicate features selected to be relevant by all three models; blue lines indicate the additional features that were selected by elastic net to be relevant.


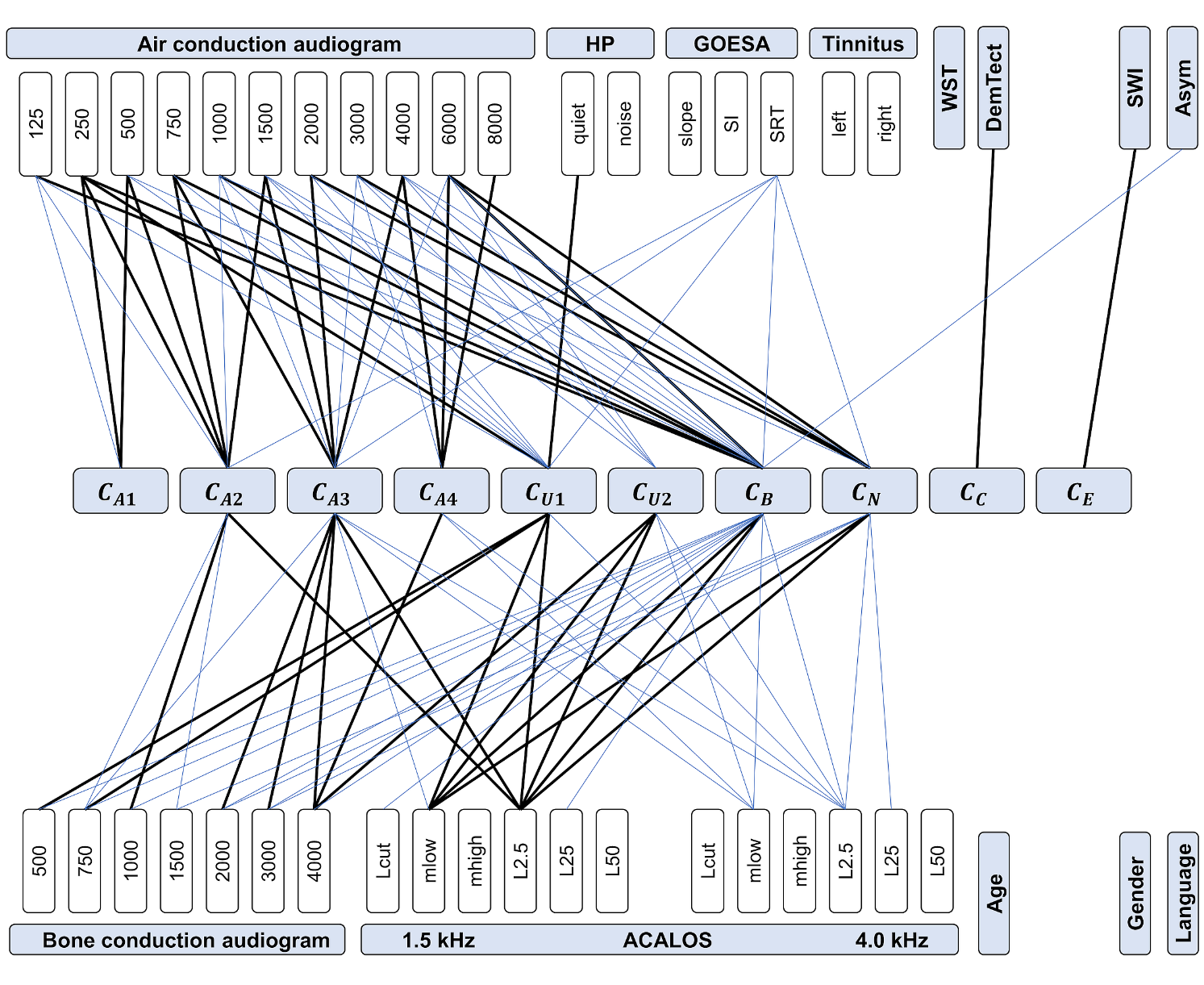


**Figure S5.** Feature importance for predicting the CAFPAs with random forests. The CAFPAs are displayed in the center of the figure; features in the upper and bottom parts. Feature parameters are represented by white boxes. Lines displayed as connections between the measures and the CAFPAs indicate features selected to be relevant for the prediction of the respective CAFPA. Black lines indicate features selected to be relevant by all three models; blue lines indicate the additional feature that were selected by random forests to be relevant.


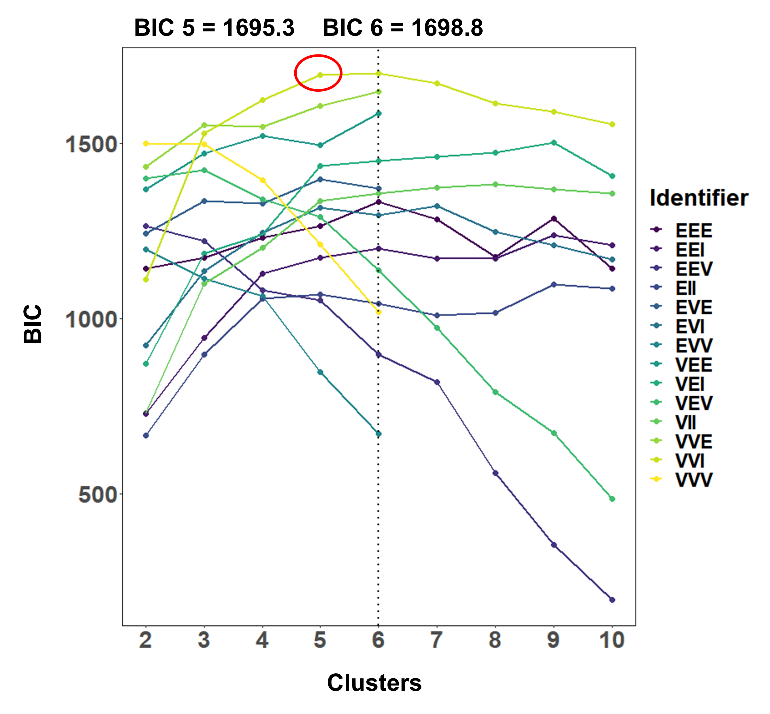
**Figure S6**. Bayesian Information Criterion (BIC) for selection of the identifier (covariance parameterization) and given number of clusters for the labeled CAFPAs. The dotted line indicates the cluster number with the maximum BIC value; the red circle the cluster number and identifier chosen for the current study. BIC 5 and BIC 6 correspond to the BIC values for five and six clusters, respectively.


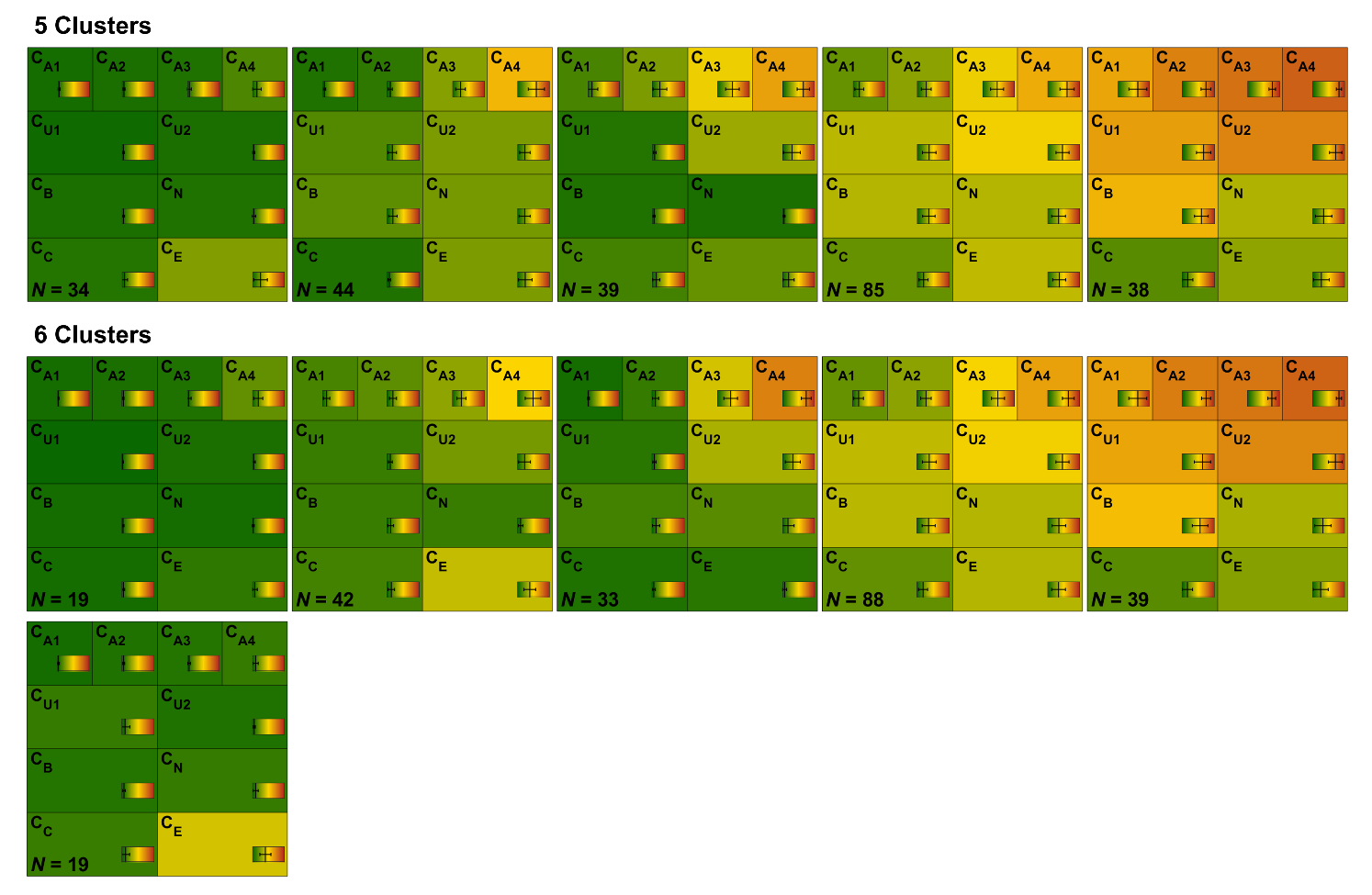


**Figure S7.** CAFPA clusters estimated using model-based clustering for the labeled patient cases, using covariance parameterization VVI. The upper part represents cluster plots for five clusters; the bottom part for six clusters. The average CAFPAs assigned to each cluster are depicted by the color of the respective area as well as by the vertical line in the color bar. Standard deviations are depicted by horizontal lines in the color bars. *N* indicates the number of patients assigned to each cluster
